# Smoking-Associated Changes in Gene Expression in Coronary Artery Disease Patients Using Matched Samples

**DOI:** 10.3390/cimb46120830

**Published:** 2024-12-07

**Authors:** Mohammed Merzah, Szilárd Póliska, László Balogh, János Sándor, Szilvia Fiatal

**Affiliations:** 1 Department of Public Health and Epidemiology, Faculty of Medicine, University of Debrecen, 4032 Debrecen, Hungary; 2 Department of Community Health, Technical Institute of Karbala, AlFurat AlAwsat Technical University, 5001 Karbala, Iraq; 3 Department of Biochemistry and Molecular Biology, Faculty of Medicine, University of Debrecen, 4032 Debrecen, Hungary; 4 Cardiology and Cardiac Surgery Clinic, University of Debrecen, 4032 Debrecen, Hungary; 5 ELKH-DE Public Health Research Group, Department of Public Health and Epidemiology, Faculty of Medicine, University of Debrecen, 4032 Debrecen, Hungary

**Keywords:** DEGs, gene expression, smoking, coronary artery disease, matched samples

## Abstract

Smoking is a well known risk factor for coronary artery disease (CAD). However, the effects of smoking on gene expression in the blood of CAD subjects in Hungary have not been extensively studied. This study aimed to identify differentially expressed genes (DEGs) associated with smoking in CAD subjects. Eleven matched samples based on age and gender were selected for analysis in this study. All subjects were non-obese, non-alcoholic, non-diabetic, and non-hypertensive and had moderate to severe stenosis of one or more coronary arteries, confirmed by coronary angiography. Whole blood samples were collected using PAXgene tubes. Next-generation sequencing was employed using the NextSeq 500 system to generate high-throughput sequencing data for transcriptome profiling. The differentially expressed genes were analyzed using the R programming language. Results: The study revealed that smokers exhibited non-significant higher levels of total cholesterol, low-density lipoprotein-cholesterol, and triglycerides compared to non-smokers (*p* > 0.05), although high-density lipoprotein-cholesterol was also elevated. Despite this, the overall lipid profile of smokers remained less favorable. Non-smokers had a higher BMI (*p* = 0.02). Differential gene expression analysis identified 58 DEGs, with 38 upregulated in smokers. The key upregulated genes included LILRB5 (log_2_FC = 2.88, *p* = 1.05 × 10^−5^) and *RELN* (log_2_FC = 3.31, *p* = 0.024), while *RNF5_2* (log_2_FC = −5.29, *p* = 0.028) and *IGHV7-4-1_1* (log_2_FC = −2.86, *p* = 0.020) were notably downregulated. Heatmap analysis showed a distinct clustering of gene expression profiles between smokers and non-smokers. However, GO analysis did not identify significant biological pathways associated with the DEGs. Conclusions: This research illuminates smoking’s biological effects, aiding personalized medicine for predicting and treating smoking-related diseases.

## 1. Introduction

Coronary artery disease (CAD) continues to be a major cause of morbidity and mortality worldwide, driven by a complex interplay of environmental, lifestyle, and genetic factors [1]. Among these, cigarette smoking is a well established risk factor for CAD, contributing to both the initiation and progression of the disease through a variety of mechanisms, including oxidative stress, endothelial dysfunction, inflammation, and lipid metabolism disturbances [2]. Despite extensive epidemiological evidence linking smoking to elevated cardiovascular risk, the precise molecular mechanisms by which smoking exacerbates CAD remain not yet fully understood [2,3].

Cardiovascular diseases (CVDs) remain a significant global health challenge, driving high rates of morbidity and mortality and underscoring the need for effective prevention strategies [4]. Traditional prevention relies on lifestyle changes, risk factor management, and medication, but advanced imaging techniques—such as ultrasound, echocardiography, cardiac MRI, and coronary CT angiography—enhance early detection, personalized risk assessment, and tailored prevention [4]. In acute myocardial infarction (AMI), where timely percutaneous coronary intervention (PCI) is critical, optical coherence tomography (OCT) optimizes outcomes by identifying lesion types and guiding PCI procedures, supporting precision medicine in CVD care [5]. Additionally, the “smoker’s paradox” highlights that smokers with STEMI may experience lower short-term mortality rates than non-smokers, potentially influenced by factors such as younger age and shorter ischemic times. However, other factors may also play a role [6].

Gene expression profiling has become a valuable method for elucidating the molecular underpinnings of complex diseases like CAD [7,8]. By pinpointing key genes, regulatory networks, and pathways that contribute to the pathophysiology of CAD, researchers hold the potential for groundbreaking insights. However, the specific effects of smoking on gene expression in CAD subjects remain a frontier waiting to be fully explored, especially within the context of matched samples. These designs, which match individuals based on key clinical or demographic variables, have the power to reduce confounding factors and illuminate the direct impact of smoking on molecular processes relevant to CAD.

Numerous studies have examined smoking-associated gene expression changes in various tissues, including peripheral blood, lung, and cardiovascular tissues [9,10,11,12]. These investigations have identified unique molecular signatures associated with smoking, including the upregulation of genes involved in inflammation, immune response, cell proliferation, and oxidative stress. However, the specific gene expression profiles associated with smoking in CAD subjects have been less thoroughly investigated [12,13]. Gaining a better understanding of these profiles could shed light on how smoking accelerates CAD progression and uncover new therapeutic targets for reducing cardiovascular risk in smokers.

Moreover, CAD is a highly heterogeneous disease, with subjects exhibiting varying levels of disease severity, clinical outcomes, and plaque composition [14]. Smoking may intensify certain pathways in some CAD subjects while having less impact on others [15]. Utilizing matched samples enables researchers to reduce patient variability and concentrate on gene expression changes directly linked to smoking in the context of CAD. This approach allows for a more refined analysis of the smoking-associated molecular signatures that play a role in the pathophysiology of CAD.

Despite the growing interest in transcriptomic studies of CAD, there remains a significant need for studies focused on how smoking influences gene expression in CAD subjects. Addressing this gap could deepen our understanding of the molecular interplay between smoking and CAD, potentially leading to personalized treatment approaches for smokers with CAD. Furthermore, identifying differentially expressed genes (DEGs) between smokers and non-smokers might uncover biomarkers for evaluating smoking-related cardiovascular risk and assessing the success of smoking cessation efforts in CAD subjects.

This study aims to investigate the gene expression changes associated with smoking in CAD subjects using matched sample designs. By comparing the transcriptional profiles of smokers and non-smokers, we seek to identify DEGs and pathways that are modulated by smoking in the context of CAD. The findings from this study could deepen our understanding of the molecular impact of smoking on CAD and pave the way for more targeted therapies and preventive measures for this high-risk patient population.

## 2. Material and Methods

**Figure 1:**
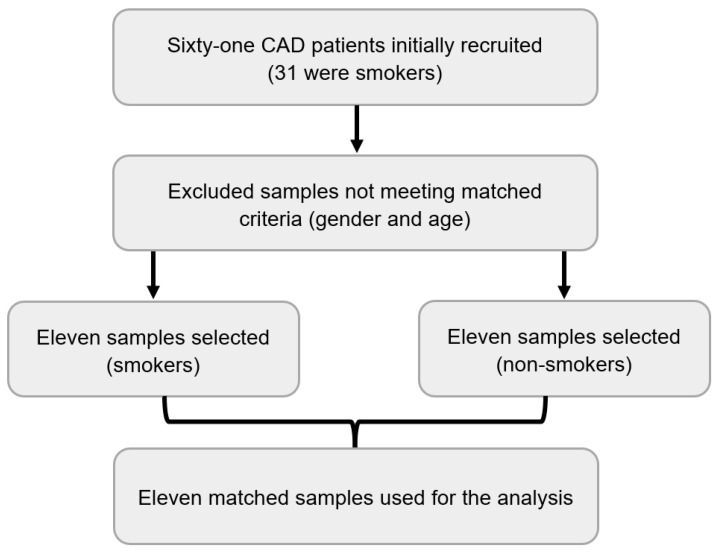
Flowchart of data selection.

The methodology for this study was detailed in our previous work [12]. Briefly, we conducted a single-center cross-sectional study at the Cardiology and Cardiac Surgery Clinic, University of Debrecen Clinical Center, between November 2021 and October 2022. Sixty-one subjects, selected based on strict inclusion (non-obese, non-alcoholic, non-diabetic, and non-hypertensive and had moderate to severe stenosis of one or more coronary arteries, confirmed by coronary angiography) were enrolled. Out of them, only 11 matched samples were selected for this analysis (Figure 1). Peripheral venous whole blood samples were collected for hematological tests, cotinine assays, and RNA analyses. RNA was extracted, quality-checked, and sequenced using the Illumina NextSeq 500 platform. Differential gene expression analysis was performed using DESeq2, and gene ontology enrichment was conducted using Cytoscape and ClueGO. Statistical analyses were carried out using R software (https://www.r-project.org/, accessed on 1 November 2024).

### 2.1. Data Preprocessing and Normalization

To prepare the RNA-Seq data for differential expression analysis, we used the DESeq2 package. Initially, raw count data were normalized to correct for variations in sequencing depth and other technical biases that could introduce discrepancies between samples. DESq2 applies a robust normalization method, adjusting for these differences to ensure accurate and unbiased comparisons between samples [16].

### 2.2. Dispersion Estimation

**Figure 2:**
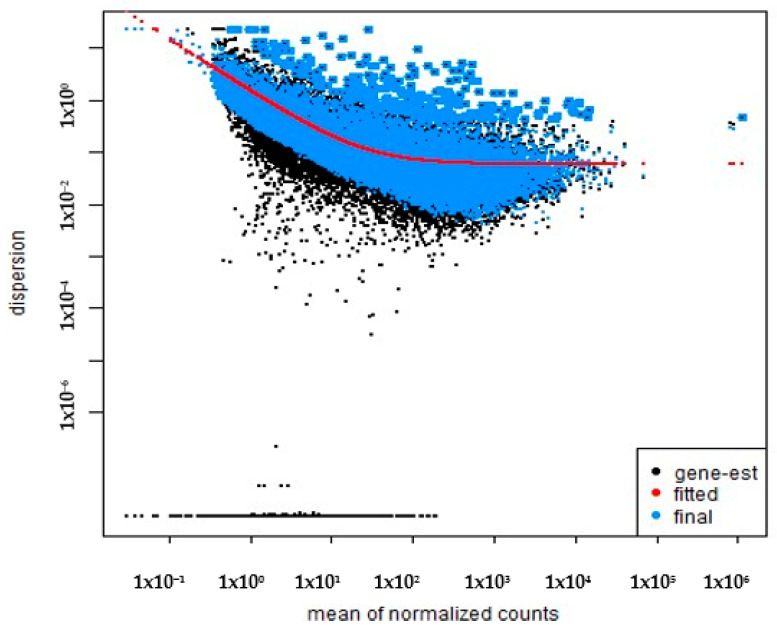
The dispersion estimates as a function of the mean of the normalized counts. The black dots represent gene-specific dispersion estimates, the blue dots correspond to the fitted values, and the red line indicates the final fitted curve.

Dispersion estimates were modeled as a function of the mean normalized counts. The relationship between the dispersion and gene expression levels is critical for adjusting the statistical model and accurately identifying DEGs. DESeq2 fits a parametric curve to the gene-wise dispersion estimates, which allows for shrinkage of the estimates towards a fitted trend, reducing noise in the data and improving statistical power [16]. The dispersion modeling is visualized in Figure 2.

## 3. Results

### 3.1. Patient Characteristics

Table 1 summarizes the baseline characteristics of the age- and gender-matched CAD subjects according to smoking status. A comparison of the two groups revealed that smokers had significantly higher levels of total cholesterol, low-density lipoprotein-cholesterol (LDL-c), and triglycerides compared to non-smokers (*p* < 0.05). However, smokers also exhibited higher levels of high-density lipoprotein-cholesterol (HDL-c). Despite the elevated HDL-c levels, the overall lipid profile was less favorable in smokers due to the more pronounced increases in total cholesterol, LDL-c, and triglycerides.

There was a significant difference in the scores for non-smokers (M = 27.5, SD = 2.0) and smokers (M = 25.0, SD = 2.7); t(12) = 2.49, *p* = 0.02. This suggests that non-smokers have a higher BMI compared to smokers. These findings suggest that the observed differences in the lipid profile may primarily be attributable to smoking status rather than confounding factors such as age, gender, or obesity.

### 3.2. Differential Expression Analysis

Following the dispersion estimates, a quantitative analysis of gene expression profiles was conducted using the moderated *t*-test to identify and compare differentially expressed genes (DEGs) between smokers and non-smokers, uncovering unique molecular signatures linked to each group. There were 58 DEGs with a FC of ≥2. Of them, 38 were upregulated. Table 2 presents the top 10 upregulated and downregulated DEGs identified when comparing smokers to non-smokers. In the upregulated category, *RELN* exhibits the highest log_2_FC (3.30) with a *p*-value of 0.027, followed by *AOX1* (log_2_FC = 2.99, *p*-value = 0.01). Notably, *LILRB5* shows a significant upregulation with a log_2_FC of 2.88, a highly significant *p*-value (1.05 × 10^−5^). Similarly, *LILRB5_2* displays a log_2_FC of 2.63, with an even lower *p*-value (1.47 × 10^−5^). It is important to note that the last two genes were kept significant even after adjusting (adjusted *p*-value of 0.05), highlighting the robustness of our findings.

On the other hand, the downregulated genes, led by *RNF5_2*, show a significant decrease with a log_2_FC of −5.29 and a *p*-value of 0.028. *RIMBP2* and *IGHV7-4-1_1* also demonstrate substantial downregulation, with log_2_FCs of −2.76 and −2.86, respectively, and respective *p*-values of 0.017 and 0.020.

**Figure 3:**
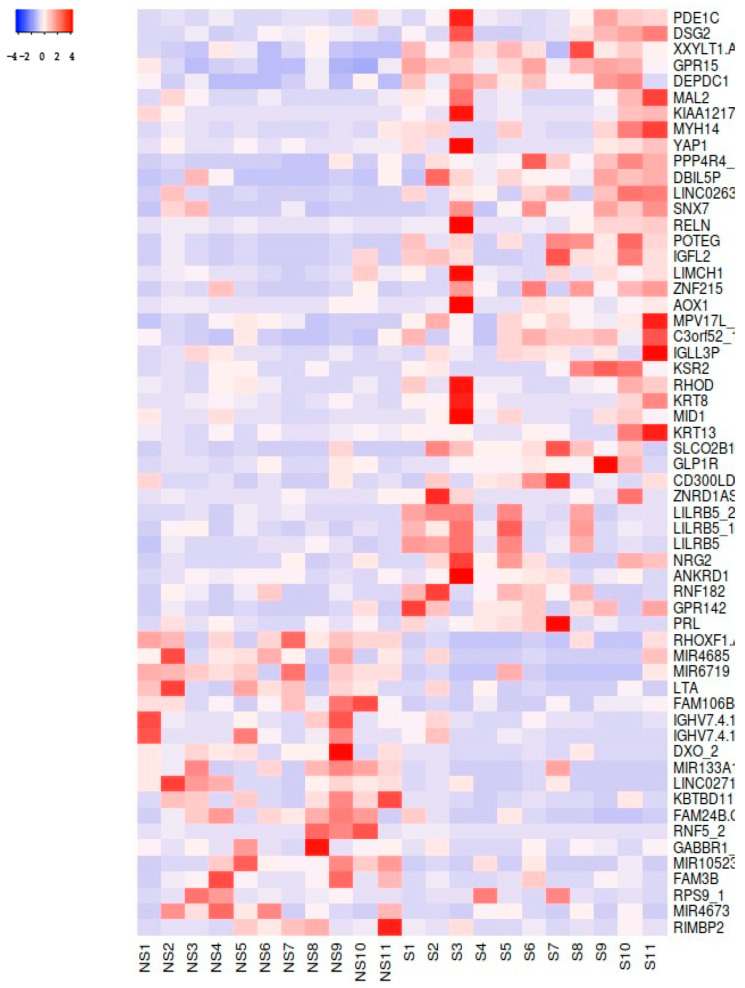
A heatmap of the 58 DEGs with a FC ≥ 2.0. S = smokers; NS = non-smokers.

Using an average linkage clustering method and Spearman Rank Correlation for distance measurement, a heatmap was generated to compare the expression profiles of the two groups in Figure 3. The heatmap clearly demonstrates distinct clustering between smokers and non-smokers, suggesting that smoking significantly impacts gene expression.

A substantial number of genes were differentially expressed, with 38 genes showing a Log_2_ FC of 2 or greater. The majority of these DEGs were upregulated in smokers compared to non-smokers, indicating a transcriptional response to smoking.

**Figure 4:**
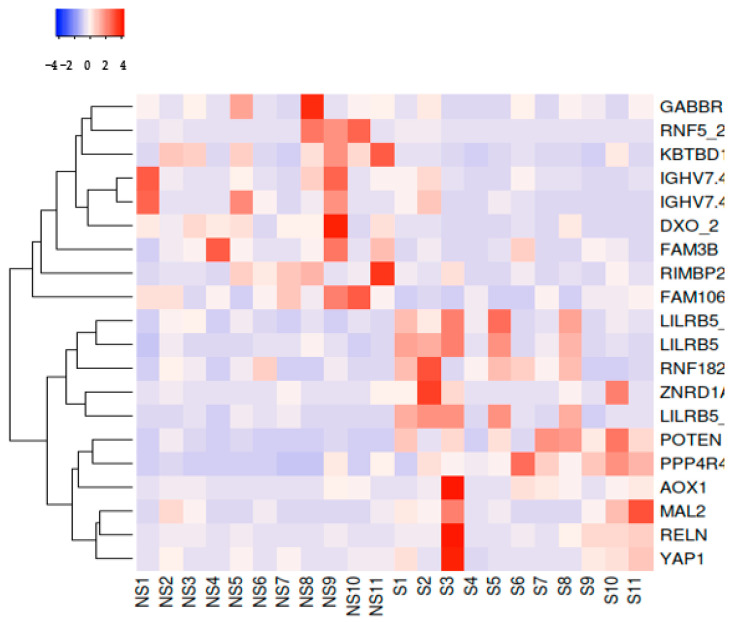
A heatmap of the top 10 upregulated and downregulated genes. S = smokers; NS = non-smokers.

Furthermore, a heatmap of the top 10 upregulated and downregulated genes was generated to emphasize the highly significant DEGs (Figure 4).

### 3.3. Gene Ontology (GO) Analysis

Although differentially expressed genes were identified, none of the GO terms or pathways showed statistical significance or meaningful biological relevance.

## 4. Discussion

Our findings suggest that smoking may be associated with an unfavorable lipid profile trend, as indicated by higher LDL-c and triglyceride levels, despite elevated HDL-c levels. Although these changes did not reach statistical significance in our sample, they align with the known adverse effects of dyslipidemia on cardiovascular health. Additionally, the identification of specific gene clusters hints at a coordinated regulatory response to smoking exposure. The individual variation observed within both groups suggests that factors beyond smoking—such as genetic predispositions or other environmental exposures—may also influence gene expression changes [17,18].

Smoking is a major risk factor for CAD, with genetic predisposition playing a significant role in modulating this relationship [19]. Studies using UK Biobank data revealed that individuals with high genetic risk experience greater absolute CAD risk due to smoking, suggesting additive gene–smoking interactions rather than multiplicative effects [20]. Genetic analyses identified shared SNPs between smoking behaviors and CAD, with significant enrichment in lipid metabolism-related pathways. Mendelian randomization highlighted that genetic liability for smoking cessation paradoxically increases CAD risk, underscoring the complex interplay between genetic factors, smoking behaviors, and CAD development [21].

Atherosclerotic CAD is influenced by both monogenic and polygenic factors [12]. Monogenic forms of CAD, though rare, are typically driven by single-gene mutations, such as those affecting *LDLR*, *PCSK9*, or *APOB*, which significantly alter lipid metabolism and accelerate atherosclerosis [22]. In contrast, the more common polygenic etiology of CAD arises from the interplay of multiple genetic variants with small individual effects, often identified through genome-wide association studies [23]. These variants interact with environmental factors and biological pathways, such as inflammation, endothelial dysfunction, and lipid metabolism, contributing to the complex and multifactorial nature of CAD [12,17].

The results reveal that smokers have significantly higher levels of total cholesterol, LDL-c, and triglycerides compared to non-smokers, although HDL-c levels are also elevated in smokers. Despite this increase in HDL-c, the overall lipid profile of smokers remains less favorable due to the elevated LDL-c and triglycerides, known risk factors for cardiovascular events. These findings are consistent with previous studies [24,25]. Conversely, non-smokers exhibited higher BMIs, which may indicate differences in lifestyle or metabolic responses between the groups.

Differential gene expression analysis identified 58 significant DEGs, with 38 upregulated in smokers. Notably, genes such as *LILRB5* (OMIM: 604814) and *AOX1* (OMIM: 602841) were highly upregulated, while *RNF5_2* (OMIM: 602677) was significantly downregulated. GO analysis highlighted distinct enrichment in processes related to skin and epithelial development in smokers, suggesting a unique transcriptional response to smoking.

Among the highly upregulated genes, *RELN* (OMIM: 600514) influences vascular endothelial cell adhesion, morphology, and membrane resistance, which are crucial for vessel wall integrity [26]. Previous research has linked elevated Reelin levels to increased vascular inflammation and atherosclerosis, suggesting that targeting Reelin could be a novel strategy for cardiovascular disease prevention [27]. *LILRB5*, involved in immune response modulation, was upregulated, indicating that smoking may impact CAD through immunological pathways [28]. This finding is consistent with emerging evidence linking smoking to inflammation and immune system dysregulation in CAD subjects [12].

Conversely, the downregulation of *RNF5_2* and genes involved in ubiquitination suggests potential alterations in protein degradation processes in smokers. Aberrant ubiquitination has been associated with various cardiovascular diseases, including atherosclerosis, which may explain the increased CAD risk in smokers [29]. The downregulation of *IGHV7-4-1* (OMIM: 147070), involved in immune response, further supports the notion of smoking-induced immune dysfunction. Smoking has been shown to reduce neutrophil phagocytic activity and interfere with immune cell functions, weakening pathogen elimination [30].

GO analysis did not reveal significant biological pathways associated with the DEGs, which may be due to the small number of DEGs, the limited representation of relevant biological processes in current GO databases, or the involvement of these genes in novel or context-specific regulatory networks. Future research using alternative pathway analysis tools or experimental validation could provide more insights into these biological mechanisms.

### Limitations

Several limitations of our study should be noted. The sample size was relatively small, which may affect the generalizability of our findings. While we identified DEGs, further validation in larger cohorts is necessary to confirm these results. Additionally, although we controlled for age and gender, other potential confounding factors, such as dietary habits, physical activity, and medication use, were not comprehensively addressed, which could have influenced the observed associations.

Our study’s cross-sectional design captures gene expression and lipid profiles simultaneously, limiting our ability to infer causality or track changes over time. Additionally, the GO analysis was constrained by the small number of DEGs and the specificity of current pathway databases, which may have hindered the identification of statistically significant pathways. Longitudinal studies could offer more insights into the dynamic nature of these changes. Furthermore, the exact mechanisms by which DEGs influence cardiovascular risk remain unclear, and functional studies are needed to elucidate their role in CAD progression.

## 5. Conclusions

Overall, our study demonstrates distinct alterations in lipid profiles and gene expression associated with smoking in CAD subjects. Specifically, we identified a significant dysregulation of lipid metabolism, immune-related pathways, and genes involved in tissue development, suggesting that smoking contributes to CAD progression through these mechanisms. These results highlight the molecular impact of smoking on CAD pathology and reinforce the critical role of smoking cessation in disease management. Additionally, the identified DEGs may serve as potential targets for therapeutic interventions. Further investigation is warranted to validate these findings in larger, more diverse cohorts and to elucidate the precise mechanistic pathways involved.

## Figures and Tables

**Table 1:** Baseline characteristics of CAD subjects.

Variables		Non-Smokers	Smokers	*p*-Value
Gender	Female	3	3	
	Male	8	8	
Age (years), mean ± SD		66 ± 6	66 ± 6	
BMI, mean ± SD		27.5 ± 2.0	25.0 ± 2.7	0.02
Cholesterol (mmol/L), mean ± SD		4.38 ± 1.21	5.25 ± 0.96	NS
Triglycerides (mmol/L), mean ± SD		1.44 ± 1.02	1.54 ± 0.44	NS
LDL-c (mmol/L), mean ± SD		2.69 ± 1.24	2.87 ± 0.92	NS
HDL-c (mmol/L), mean ± SD		1.09 ± 0.20	1.66 ± 1.13	NS
Plasma cotinine ng/mL, mean ± SD		0.4 ± 0.2	0.2 ± 0.1	NS

BMI = body mass index, HDL-c = high-density lipoprotein cholesterol, LDL-c = low-density lipoprotein cholesterol, NS = no significant, SD = standard deviation.

**Table 2:** The top 10 up and downregulated differentially expressed genes in smokers vs. non-smokers.

Gene ID	Gene Name	log_2_FC	*p*-Value
Upregulated			
*RELN*	Reelin	3.31	0.024
*AOX1*	Aldehyde Oxidase 1	2.99	0.015
*ZNRD1ASP_6*	Zinc Ribbon Domain Containing 1 Antisense RNA (variant 6)	2.98	0.023
*LILRB5*	Leukocyte Immunoglobulin Like Receptor B5	2.88	1.05 × 10^−5^
*YAP1*	Yes Associated Protein 1	2.86	0.012
*RNF182*	Ring Finger Protein 182	2.77	0.008
*PPP4R4_1*	Protein Phosphatase 4 Regulatory Subunit 4 (variant 1)	2.64	0.002
*LILRB5_2*	Leukocyte Immunoglobulin Like Receptor B5 (variant 2)	2.63	1.47 × 10^−5^
*MAL2*	Mal, T-cell differentiation protein 2	2.63	0.007
*LILRB5_1*	Leukocyte Immunoglobulin-Like Receptor Subfamily B Member 5, isoform 1	2.57	5.83 × 10^−5^
Downregulated			
*RNF5_2*	Ring Finger Protein 5 (variant 2)	−5.29	0.028
*IGHV7-4-1_1*	Immunoglobulin Heavy Variable 7-4-1 (variant 1)	−2.86	0.020
*RIMBP2*	RIMS Binding Protein 2	−2.76	0.017
*IGHV7-4-1*	Immunoglobulin Heavy Variable 7-4-1	−2.57	0.042
*DXO_2*	Decapping And Exoribonuclease (variant 2)	−2.57	0.014
*POSTN*	Periostin	−2.53	0.049
*KBTBD11-AS1_1*	KBTBD11 Antisense RNA 1 (variant 1)	−2.36	0.033
*FAM3B*	Family With Sequence Similarity 3 Member B	−2.31	0.006
*FAM106B*	Family with Sequence Similarity 106, Member B	−2.20	0.009
*GABBR1_2*	Gamma-aminobutyric acid type B receptor	−2.16	0.038

Bold font indicates significant adjusted *p*-value.

## Data Availability

The data presented in this study are available on request from the corresponding author. The data are not publicly available due to ethical restrictions.

## References

[1] Amit V.K., Sekar K. (2017). Genetics of coronary artery disease: Discovery, biology and clinical translation. Nat. Rev. Genet..

[2] Gallucci G., Tartarone A., Lerose R., Lalinga A.V., Capobianco A.M. (2020). Cardiovascular risk of smoking and benefits of smoking cessation. J. Thorac. Dis..

[3] Klein L.W. (2022). Pathophysiologic Mechanisms of Tobacco Smoke Producing Atherosclerosis. Curr. Cardiol. Rev..

[4] Trimarchi G., Pizzino F., Paradossi U., Gueli I.A., Palazzini M., Gentile P., Di Spigno F., Ammirati E., Garascia A., Tedeschi A. (2024). Charting the Unseen: How Non-Invasive Imaging Could Redefine Cardiovascular Prevention. J. Cardiovasc. Dev. Dis..

[5] Buonpane A., Trimarchi G., Ciardetti M., Coceani M.A., Alagna G., Benedetti G., Berti S., Andò G., Burzotta F., De Caterina A.R. (2024). Optical Coherence Tomography in Myocardial Infarction Management: Enhancing Precision in Percutaneous Coronary Intervention. J. Clin. Med..

[6] Paradossi U., De Caterina A.R., Trimarchi G., Pizzino F., Bastiani L., Dossi F., Raccis M., Bianchi G., Palmieri C., de Gregorio C. (2024). The enigma of the ‘smoker’s paradox’: Results from a single-center registry of patients with STEMI undergoing primary percutaneous coronary intervention. Cardiovasc. Revasc. Med..

[7] Hartiala J., Schwartzman W.S., Gabbay J., Ghazalpour A., Bennett B.J., Allayee H. (2017). The Genetic Architecture of Coronary Artery Disease: Current Knowledge and Future Opportunities. Curr. Atheroscler. Rep..

[8] Björkegren J.L.M., Kovacic J.C., Dudley J.T., Schadt E.E. (2015). Genome-wide significant loci: How important are they? Systems genetics to understand heritability of coronary artery disease and other common complex disorders. J. Am. Coll. Cardiol..

[9] Maas S.C.E., Mens M.M., Kühnel B., van Meurs J.B., Uitterlinden A.G., Peters A., Prokisch H., Herder C., Grallert H., Kunze S. (2020). Smoking-related changes in DNA methylation and gene expression are associated with cardio-metabolic traits. Clin. Epigenetics.

[10] Paul S., Amundson S.A. (2014). Differential Effect of Active Smoking on Gene Expression in Male and Female Smokers. J. Carcinog. Mutagen..

[11] Arimilli S., Makena P., Liu G., Prasad G.L. (2019). Distinct gene expression changes in human peripheral blood mononuclear cells treated with different tobacco product preparations. Toxicol. Vitr..

[12] Merzah M., Póliska S., Balogh L., Sándor J., Szász I., Natae S., Fiatal S. (2023). A Transcriptomic Analysis of Smoking-Induced Gene Expression Alterations in Coronary Artery Disease Patients. Int. J. Mol. Sci..

[13] Nowak J.K., Dybska E., Adams A.T., Walkowiak J. (2022). Immune cell-specific smoking-related expression characteristics are revealed by re-analysis of transcriptomes from the CEDAR cohort. Cent. J. Immunol..

[14] Achim A., Péter O., Cocoi M., Serban A., Mot S., Dadarlat-Pop A., Nemes A., Ruzsa Z. (2023). Correlation between Coronary Artery Disease with Other Arterial Systems: Similar, Albeit Separate, Underlying Pathophysiologic Mechanisms. J. Cardiovasc. Dev. Dis..

[15] Salehi N., Janjani P., Tadbiri H., Rozbahani M., Jalilian M. (2021). Effect of cigarette smoking on coronary arteries and pattern and severity of coronary artery disease: A review. J. Int. Med. Res..

[16] Li X., Cooper N.G.F., O’Toole T.E., Rouchka E.C. (2020). Choice of library size normalization and statistical methods for differential gene expression analysis in balanced two-group comparisons for RNA-seq studies. BMC Genom..

[17] Natae S.F., Kósa Z., Sándor J., Merzah M.A., Bereczky Z., Pikó P., Ádány R., Fiatal S. (2021). The higher prevalence of venous thromboembolism in the Hungarian Roma population could be due to elevated genetic risk and stronger gene-environmental interactions. Front. Cardiovasc. Med..

[18] Hassan S.H., Merzah M.A. (2019). Study on The Effect of Cigarette Smoke on Human Health. Biochem. Cell. Arch..

[19] Ramírez J., van Duijvenboden S., Young W.J., Tinker A., Lambiase P.D., Orini M., Munroe P.B. (2022). Prediction of coronary artery disease and major adverse cardiovascular events using clinical and genetic risk scores for cardiovascular risk factors. Circ. Genom. Precis. Med..

[20] Huang Y., Hui Q., Gwinn M., Hu Y., Quyyumi A.A., Vaccarino V., Sun Y.V. (2022). Interaction between genetics and smoking in determining risk of coronary artery diseases. Genet. Epidemiol..

[21] Zhu Z., Liu Q., Li M., Yao Y., Qi F., Xu Y., Lu S., Yang Z., Guan Y., Li M.D. (2023). Determination of genetic correlation between tobacco smoking and coronary artery disease. Front. Psychiatry.

[22] Merzah M., Natae S., Sándor J., Fiatal S. (2024). Single Nucleotide Variants (SNVs) of the Mesocorticolimbic System Associated with Cardiovascular Diseases and Type 2 Diabetes: A Systematic Review. Genes.

[23] Kessler T., Schunkert H. (2021). Coronary Artery Disease Genetics Enlightened by Genome-Wide Association Studies. JACC Basic Transl. Sci..

[24] Nakamura M., Yamamoto Y., Imaoka W., Kuroshima T., Toragai R., Ito Y., Kanda E., Schaefer E.J., Ai M. (2021). Relationships between Smoking Status, Cardiovascular Risk Factors, and Lipoproteins in a Large Japanese Population. J. Atheroscler. Thromb..

[25] Mambo A., Yang Y., Mahulu E., Zihua Z. (2024). Investigating the interplay of smoking, cardiovascular risk factors, and overall cardiovascular disease risk: NHANES analysis 2011–2018. BMC Cardiovasc. Disord..

[26] Alexander A., Herz J., Calvier L. (2023). Reelin through the years: From brain development to inflammation. Cell Rep..

[27] Ding Y., Huang L., Xian X., Yuhanna I.S., Wasser C.R., Frotscher M., Mineo C., Shaul P.W., Herz J. (2016). Loss of Reelin protects against atherosclerosis by reducing leukocyte-endothelial cell adhesion and lesion macrophage accumulation. Sci. Signal..

[28] Hogan L.E., Jones D.C., Allen R.L. (2016). Expression of the innate immune receptor LILRB5 on monocytes is associated with mycobacteria exposure. Sci. Rep..

[29] Kong P., Cui Z.-Y., Huang X.-F., Zhang D.-D., Guo R.-J., Han M. (2022). Inflammation and atherosclerosis: Signaling pathways and therapeutic intervention. Signal Transduct. Target. Ther..

[30] Hecht S.S. (2003). Tobacco carcinogens, their biomarkers and tobacco-induced cancer. Nat. Rev. Cancer.

